# Explore potential disease related metabolites based on latent factor model

**DOI:** 10.1186/s12864-022-08504-w

**Published:** 2022-04-06

**Authors:** Yongtian Wang, Liran Juan, Jiajie Peng, Tao Wang, Tianyi Zang, Yadong Wang

**Affiliations:** 1 School of Computer Science, Northwestern Polytechnical University, Xi’an, China; 2 Key Laboratory of Big Data Storage and Management Ministry of Industry and Information Technology, Northwestern Polytechnical University, Xi’an, China; 3 School of Life Science and Technology, Harbin Institute of Technology, Harbin, China; 4 School of Computer Science and Technology, Harbin Institute of Technology, Harbin, China

**Keywords:** Metabolite, Disease similarity, Disease diagnosis, Matrix decomposition

## Abstract

**Background:**

In biological systems, metabolomics can not only contribute to the discovery of metabolic signatures for disease diagnosis, but is very helpful to illustrate the underlying molecular disease-causing mechanism. Therefore, identification of disease-related metabolites is of great significance for comprehensively understanding the pathogenesis of diseases and improving clinical medicine.

**Results:**

In the paper, we propose a disease and literature driven metabolism prediction model (DLMPM) to identify the potential associations between metabolites and diseases based on latent factor model. We build the disease glossary with disease terms from different databases and an association matrix based on the mapping between diseases and metabolites. The similarity of diseases and metabolites is used to complete the association matrix. Finally, we predict potential associations between metabolites and diseases based on the matrix decomposition method. In total, 1,406 direct associations between diseases and metabolites are found. There are 119,206 unknown associations between diseases and metabolites predicted with a coverage rate of 80.88%. Subsequently, we extract training sets and testing sets based on data increment from the database of disease-related metabolites and assess the performance of DLMPM on 19 diseases. As a result, DLMPM is proven to be successful in predicting potential metabolic signatures for human diseases with an average AUC value of 82.33%.

**Conclusion:**

In this paper, a computational model is proposed for exploring metabolite-disease pairs and has good performance in predicting potential metabolites related to diseases through adequate validation. The results show that DLMPM has a better performance in prioritizing candidate diseases-related metabolites compared with the previous methods and would be helpful for researchers to reveal more information about human diseases.

## Background

It is an important challenge to reveal the relationship between disease phenotype and potential cell dysfunction in the biomedicine field [1–3]. In the past decades, people have been working on gene-based methods to identify specific genetic defects. However, most cell components perform their functions through complex networks involving gene regulation, metabolism and protein–protein interaction. Although these methods have made great progress in disease treatment, it is still far from enough [2, 4, 5]. In clinical practice, metabolites are often used as biological indicators for disease diagnosis [6]. For example, people have been using small amounts of metabolites to assess individual health, such as glucose, cholesterol, creatinine, urea and so on. A large number of metabolites are also used as biomarkers for the diagnosis and treatment of congenital metabolic defects [7]. However, in the face of diseases caused by multiple factors such as type 2 diabetes, metabolic syndrome or neurodegenerative disease, clinical diagnosis and treatment urgently need more types of biomarkers [6].

Metabonomics, an important part of system biology, is a way to analyze metabolites quantitatively and identify the relationship between metabolites and physiological and pathological changes. The emergence of metabonomics has improved our understanding of intracellular metabolites [8]. In addition, in all omics, it is considered to be closer to the biological phenotypes, so metabonomics is an effective approach to study them [9, 10]. For example, metabonomics can be used as a powerful tool for human precision medicine [11]. Some researchers have selected 80 healthy volunteers for metabonomics research. The results show that the changes of metabolites are individual specific and related to genetic changes, which can be used for disease risk assessment [12]. Therefore, studying the interaction between metabolites and disease phenotypes can help people understand more about the regulatory networks in organisms.

In a metabolic network, a metabolite is not only associated with a sole disease, but also with a variety of diseases [13]. Therefore, the adjacent metabolites with functional associations are more likely to be related to the same or similar diseases [2]. This suggests that functional associations between metabolites can be measured by disease similarity. This paper aims to identify more potential disease-related metabolites by analyzing metabolites and disease data, and propose a disease-related metabolite prediction method integrating disease and literature based on latent factor model.

The contribution of this paper is mainly shown in the following aspects:

- A disease vocabulary is built, by which can further expand the application of the disease ontology.
- The metabolite-related disease similarity and the literature associations of metabolites are concurrently considered. It can better reflect the relationship between metabolites and diseases.
- Using the disease and metabolite similarity to identify the unknown association between them can effectively avoid the problem of data sparsity and improve the prediction accuracy of disease-related metabolites combining with the matrix decomposition method.

## Results

**Fig. 1:**
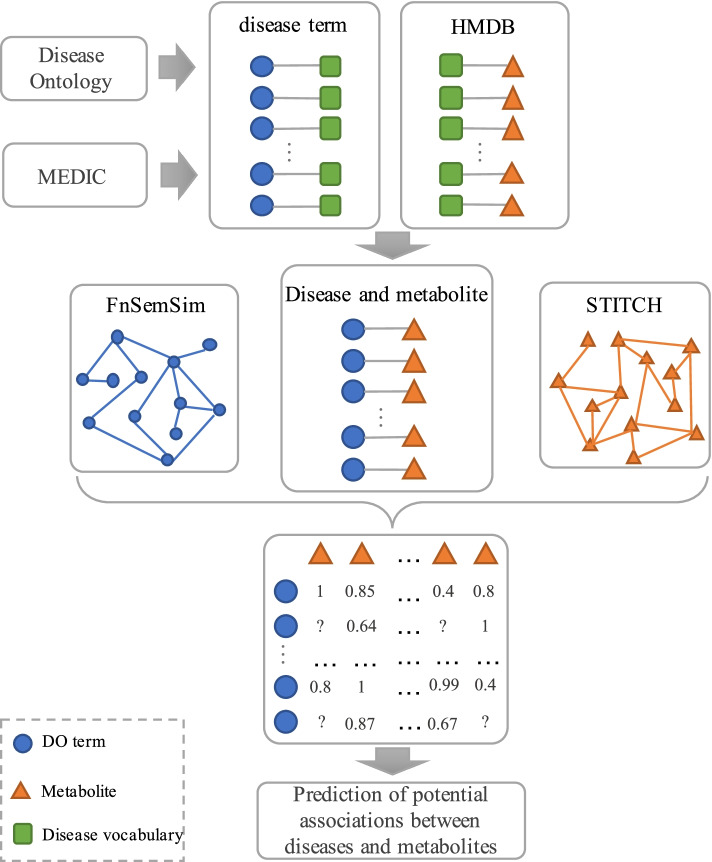
Schematic of metabolite prediction based on disease and literature association

In the paper, we propose a disease and literature driven metabolism prediction model (DLMPM) to identify the potential associations between metabolites and diseases based on latent factor model. The workflow of the computational model is shown in Fig. 1.

The disease and literature driven metabolism prediction model (DLMPM) is proposed to predict the association between diseases and metabolites by using the known disease similarity and the related metabolite literature correlation scores. Firstly, the disease terms are extracted from the experimental data that is preprocessed. A disease vocabulary is constructed by the synonym mapping between the disease terms. Then, according to the disease vocabulary and the associations between known diseases and metabolites, the mapping between disease ontology items and metabolites is established; Based on the mapping, the unknown associations between diseases and metabolites are identified by using the disease similarity and the literature association score of metabolites, and a predictive association matrix of diseases and metabolites is constructed. Finally, the metabolites related to diseases are classified by matrix decomposition method and the metabolites potentially associated with disease are predicted.

### Metabolites and diseases

A total of 8,704 DO terms are integrated and a disease vocabulary containing 68,838 terms is built. Then, 1,406 associations between metabolites and diseases are found by using the disease vocabulary and the mappings between metabolites and diseases provided, including 248 disease terms and 600 metabolites.

The literature association scores between metabolites from STITCH are extracted and taken as the metabolite similarities. Finally, 27,558 associations of 492 metabolites are obtained from STITCH. Meanwhile, a total of 37,846 associations between 229 diseases are obtained when the disease similarities are calculated.

**Fig. 2:**
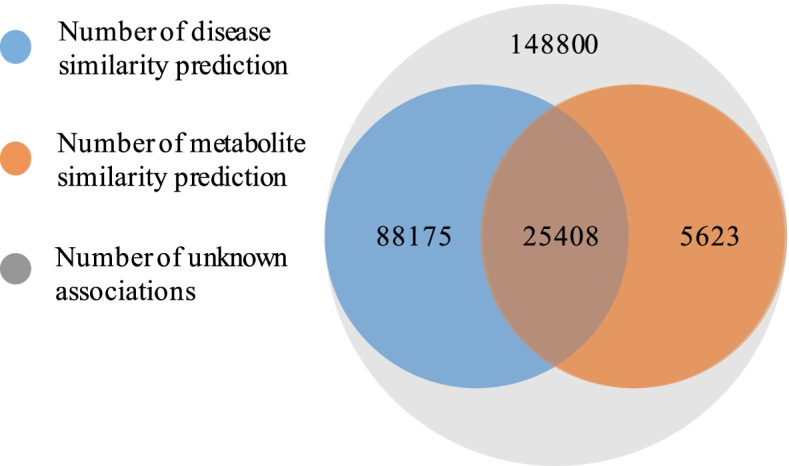
Distribution of the predictive associations between diseases and metabolites

In total, there are 1,406 direct associations between diseases and metabolites. On this basis, the similarity calculation of diseases and metabolites is used to predict the unknown associations between diseases and metabolites in the relational matrix. There are 119,206 unknown associations between diseases and metabolites predicted with a coverage rate of 80.88%. The distribution of the predicted associations based on disease similarity and metabolite similarity is shown in Fig. 2. When the unknown associations in the matrix are predicted, the number of predicted associations based on both disease and metabolite similarity is 25,408. It is 88,175 when the associations predicted by the disease similarity and the number when the metabolite similarity used is 5,623.

### Performance

In the previous study [14], we designed a validation scheme to extract test sets based on data increment from the database of disease-related metabolites. Data increment means that the volume and quality of the data in the disease-related metabolite database continue to expand and improve because the data is updated regularly. So the differences between the versions of the databases can be used to collect test data. Here the formal definition of the test set is given as follows:

*Definition 3 Existing a bipartite graph MDG *= *(M, D, MAP), where M is the collection of metabolites and D is the collection of diseases, Map: MD is the collection of associations between diseases and metabolites. Given MDG*_*1*_* and MDG*_*2*_*, for ∀d ∈ D*_*1*_*, if ∃m ∈ M*_*1*_* ∩ M*_*2*_, satisfying (m → d) ∈ Map1 and (m → d) ∈ Map2, then metabolite m is one of detection targets for disease d. For ∀ m^*^ ∈ M_*1*_* ∩ M*_*2*_, satisfying (m^*^ → d) ∈ Map2, then metabolite m^*^ is a positive example in the test set of disease d.

**Fig. 3:**
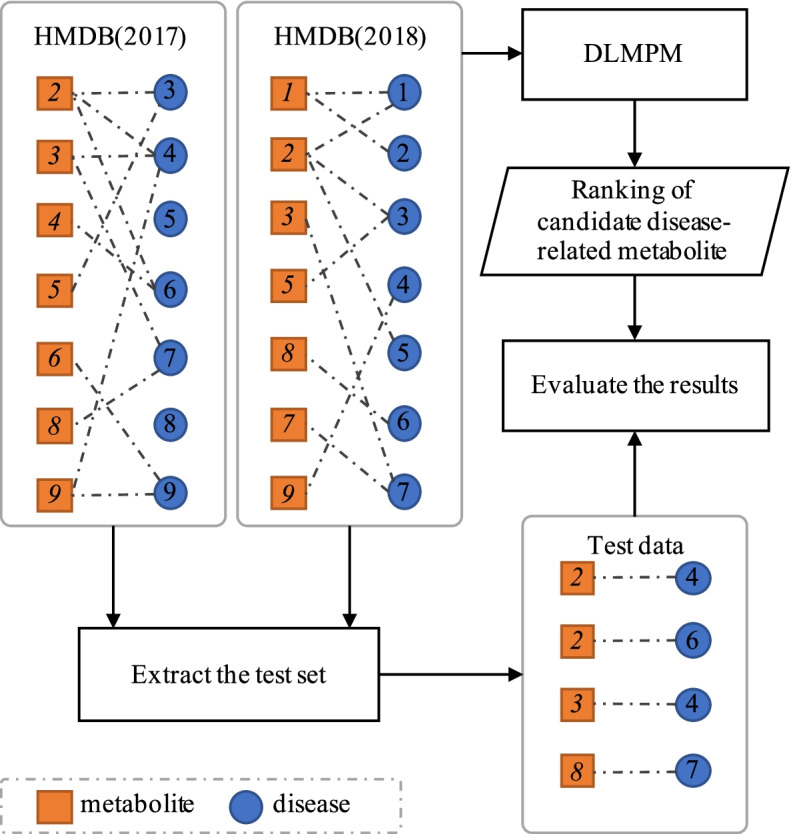
The validation scheme of DLMPM

The associations between metabolites and diseases can be seen as a bipartite graph. According to Definition 3, test data can be extracted to validate the prediction model. Specifically, the prediction method is firstly performed by using the data in the version 2017 of HMDB and then the test data can be extracted based on the version 2018. The validation process is shown in Fig. 3. By comparing the different versions of the data, 19 diseases meet the conditions for validation. The 19 diseases and their related metabolites are used to assess the performance of predicting disease-associated metabolites. The average AUC value of DLMPM reaches 82.33%, indicating that the model for predicting potential disease-related metabolites proposed in this paper have a good performance to identify potential associations between diseases and metabolites.

**Fig. 4:**
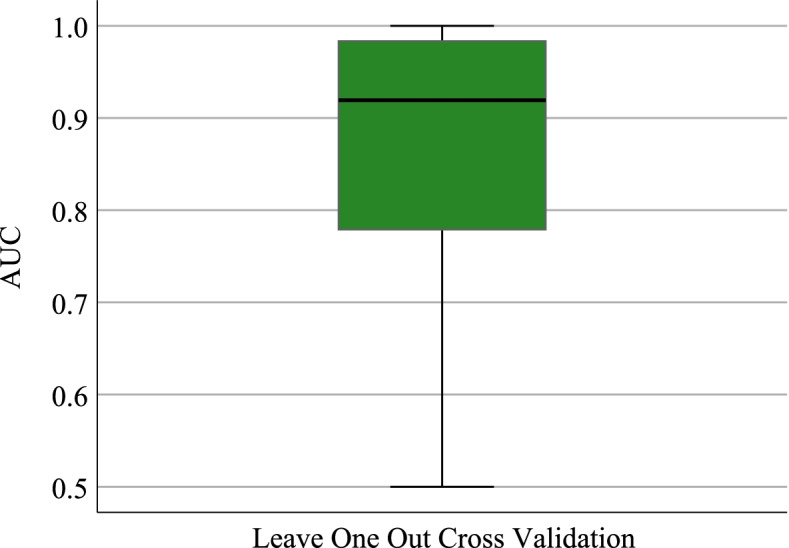
Performance of DLMPM with LOOCV

In addition, Leave One Out Cross Validation (LOOCV) is used to further validate the generalization ability of DLMPM for predicting disease-related metabolites. Specifically, after removing any association between a disease and a metabolite, DLMPM is built based on the other known associations and then validated with the removed one. We performed LOOCV for each pair of a disease and a metabolite and the average AUC of DLMPM can reach 86.83%, as shown in Fig. 4. It indicates that DLMPM has a good generalization ability for exploring potential disease related metabolites.

## Discussion

**Fig. 5:**
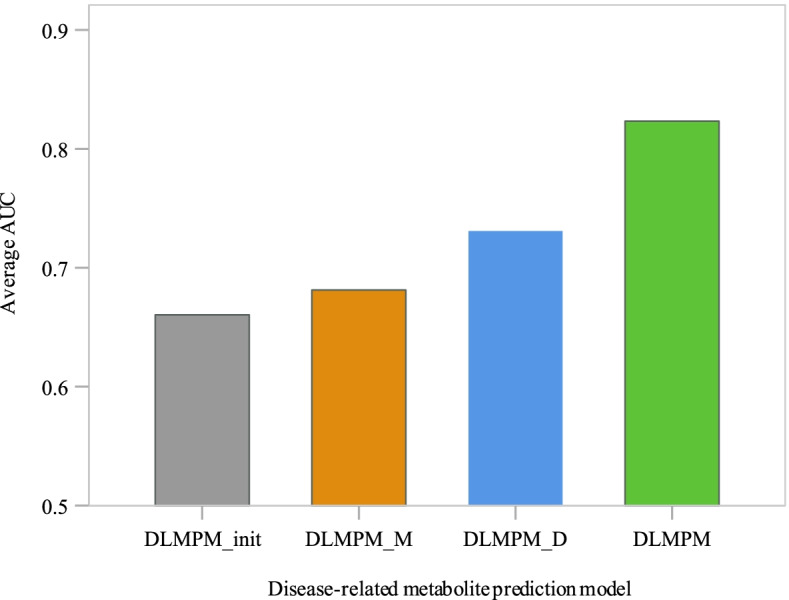
Average AUC of the metabolite prediction models

DLMPM uses the similarity of diseases and metabolites to complete the association matrix for the disease-related metabolite prediction, so an experiment is designed to verify the necessity of introducing disease and metabolite similarity. We build DLMPM_init based on direct associations between diseases and metabolites, DLMPM_D based on the disease similarity, DLMPM_M based on the metabolite similarity. Then the test set from these 19 diseases is used to validate these prediction models as shown in Fig. 5. DLMPM_init can effectively predict the potential association between metabolites and diseases with an average AUC value of 66.04%. The performance of DLMPM_M is better than DLMPM_init and reaches 68.12%. Based on the disease similarity, DLMPM_D has an average AUC value of 73.08%. Compared with these methods, the prediction ability of DLMPM is greatly improved and its average AUC reaches 82.33%.

**Fig. 6:**
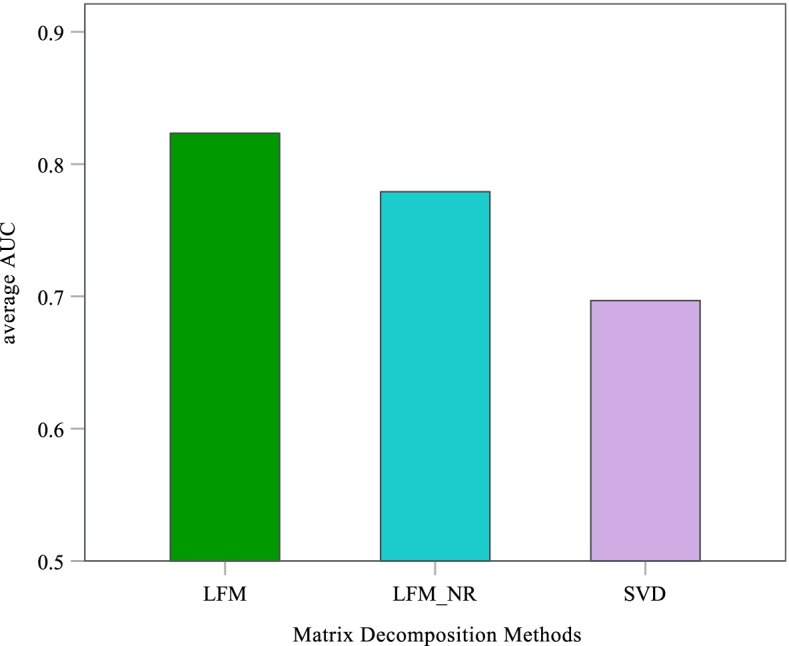
Average AUC of prediction models based on different matrix decomposition methods

In addition, we compare the differences of prediction results with different matrix decomposition methods. Here we implement SVD-based recommendation algorithm and LFM_NR method. SVD-based method uses matrix decomposition to obtain feature vectors and predicts the associations between diseases and metabolites based on dimensionality reduction data. LFM_NR is similar to LFM in principle, except that there is no regularization in the optimization function. Then the test scheme based on data increment is used to validate these prediction models as shown in Fig. 6. SVD method can effectively predict the potential association between metabolites and diseases with an average AUC value of 69.69%. The performance of LFM_NR is better than SVD and reaches 77.91%. It is clear that the predictive power of DLMPM is outstanding compared with these methods.

**Fig. 7:**
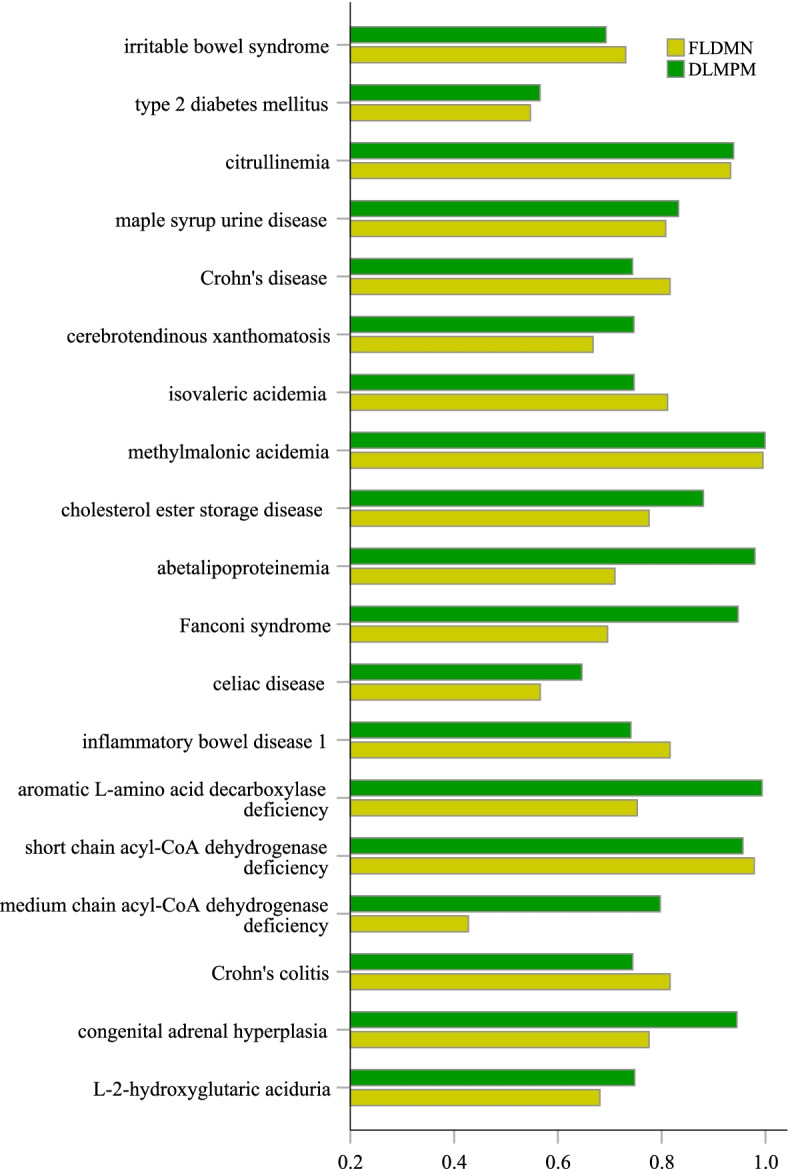
Performances of the metabolite prediction models based on 19 diseases

We compare DLMPM with the existing method FLDMN [14] that has proposed to predict potential disease-related metabolites. In FLDMN, a spatial vector with disease as the dimension was used to calculate the similarity of metabolites and a model-based collaborative filtering algorithm was used to predict disease-related metabolites. We use the two prediction models to predicting potential metabolites associated with these 19 diseases. As shown in Fig. 7, DLMPM has a better performance than FLDMN in identifying disease-related metabolites. The predictive power for 13 of these diseases is improved significantly. For example, for disease “Fanconi syndrome” (DOID:1062), the average AUC of FLDMN is 69.58% while DLMPM has an average AUC of 94.72%. In addition, the average AUC of the DLMPM-based prediction model for 16 diseases is more than 70% and more than 90% for 7 diseases. By comparison, the performance of FLDMN for 13 diseases is more than 70% and more than 90% for 3 diseases. In general, the average AUC of FLDMN is 76.03% and the AUC of DLMPM can reach 82.33%, which has a better performance in predicting potential associations between diseases and metabolites.

We found that DLMPM has outstanding ability to predict metabolites associated with certain diseases. For example, the average AUC reaches 99.32% based on DLMPM for disease “aromatic L-amino acid decarboxylase deficiency” (DOID:0,090,123). Similarly, the AUC value for disease “methylmalonic acidemia” (DOID:14,749) is also more than 99%. But the experimental results show that DLMPM is not capable of predicting certain disease. For disease “type 2 diabetes mellitus” (DOID:0,090,123), the AUC values of DLMPM and FLDMN are less than 60%. The lack of recognition ability for this disease is related to the known associations between diseases and metabolites collected in the process of building the prediction model. The previous study has shown that metabolites associated with “type 2 diabetes mellitus” are different in different versions of HMDB. The difference is that the number of candidate metabolites to be identified is 1, but the number of disease-related metabolites in different versions of HMDB is the same. The detailed data can be found in table 1 of the literature [14]. Therefore, it can be seen as a correction for disease-related metabolites and the prediction based on incorrect information may affect the result.

### Case study

We use several diseases as examples to predict potential associations between them and candidate metabolites by DLMPM based on the latest data from HMDB. Alzheimer's disease (DOID:10,652) is a neurodegenerative disease characterized by memory impairment, aphasia and executive dysfunction. The etiology of Alzheimer's disease is still unknown. In the list of predicted metabolites for Alzheimer's disease, nine of the top ten metabolites are highly associated with Alzheimer's disease according to the literature, but they have not been included in HMDB. The specific information can be seen in Table 1. For example, the study [15] has discussed the role of hydrogen peroxide (HMDB0003125) in the aetiology of Alzheimer's disease. The toxicity of the H_2_O_2_ molecule may be closely linked with the role of heavy metals in Alzheimer’s disease pathology.

**Table 1:** Prediction of potential related metabolites with complex diseases

Disease	Metabolite Term	HMDB ID	Ranking	Evidence
Alzheimer's disease	Adenosine triphosphate	HMDB0000538	1	Ref [16]
	Ethanol	HMDB0000108	2	Ref [17]
	L-methionine	HMDB0000696	3	Ref [18]
	Ammonia	HMDB0000051	4	Ref [19]
	Hydrogen peroxide	HMDB0003125	5	Ref [15]
	Sucrose	HMDB0000258	6	Ref [20]
	Uric acid	HMDB0000289	8	Ref [21]
	Norepinephrine	HMDB0000216	9	Ref [22]
	Guanosine triphosphate	HMDB0001273	10	Ref [23]
Breast cancer	L-Arginine	HMDB0000517	3	Ref [24]
	Glycine	HMDB0000123	4	Ref [25, 26]
	L-Lysine	HMDB0000182	5	Ref [27]
Type 1 diabetes mellitus	L-Arginine	HMDB0000517	3	Ref [28]
	Ethanol	HMDB0000108	4	Ref [29]

We also use DLMPM to predict candidate metabolites for type 1 diabetes mellitus (DOID:9744) and breast cancer (DOID:1612) respectively. L-Arginine (HMDB0000517) and Ethanol (HMDB0000108) are ranked in the top five of candidate metabolites for type 1 diabetes mellitus. Their associations with type 1 diabetes mellitus have been documented [28, 29]. In the list of candidate metabolites for breast cancer, L-Arginine (HMDB0000517), Glycine (HMDB0000123) and L-Lysine (HMDB0000182) are in the top five, while there have been several studies on their roles in breast cancer research [24–27]. For examples, researchers have found that L-arginine can stimulate host defenses in patients with breast cancer.

## Conclusions

Metabolite, as the link between genotypes and phenotypes, can be used to explain the underlying molecular disease-causing mechanisms. Therefore, we propose a novel prediction method DLMPM to identify candidate metabolites related to diseases based on latent factor model. We first build the disease glossary with disease ontology and MeSH and establish the mapping between diseases and metabolites. The unknown elements in the association matrix of diseases and metabolites are filled with the similarity of diseases and metabolites. Finally, we predict potential associations between metabolites and diseases based on the matrix decomposition method. The result shows that DLMPM is proved successful in predicting novel metabolic signatures with an average AUC value of 82.33%. Compared with the previous method, DLMPM has been greatly improved and would be helpful for researchers in metabolomics.

## Methods

### Data integration

Human Metabolome Database(HMDB) is a standard metabolomic resource containing detailed information about small molecule metabolites found in the human body [30]. The disease information related to human metabolites can be extracted from the XML file provided by HMDB. However, there is no uniform representation of disease names in the extracted information. It hinders the establishment of mappings between diseases and metabolites because the correspondence among different disease names cannot be determined. Therefore, it is necessary to establish a glossary rich in disease vocabulary and then annotate the disease terms with it. In this study, there are two disease data sources for the disease term integration: Disease Ontology(DO) [31] and MEDIC [32]. DO, as a standardized human disease ontology, provides a unified description of disease terminology for biomedicine, including human disease terminology, phenotypic characteristics and disease-related medical concepts. By cross-mapping with MeSH [33], ICD [34], NCI Thesaurus [35], SNOMED [36] and OMIM [37], DO integrates a large number of diseases and medical vocabulary semantically. MEDIC is a disease vocabulary maintained by CTD [38]. It contains more than 9,700 diseases and more than 67,000 disease terms and synonymous descriptions. Although MEDIC is not a medical ontology, it plays a huge role in establishing links of diseases and toxicological genomics. MEDIC integrates the disease terms from OMIM in accordance with the disease hierarchy of MeSH.

**Fig. 8:**
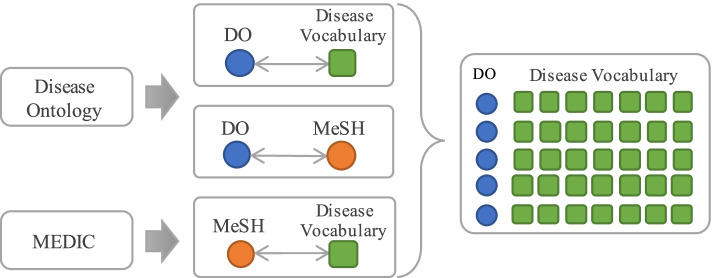
Schematic of expanding disease terms

Because both DO and MEDIC contain a large number of disease words with the same meaning, disease synonyms can be extracted from DO and MEDIC respectively. Based on the mapping of diseases in DO and MeSH provided by DO, disease synonyms are used to annotate disease terms in DO. In this way, the disease vocabulary can be expanded, as shown in Fig. 8.

Finally, 82,921 terms in MEDIC were annotated into DO and the vocabulary of DO is expanded by 45,495 items based on the mapping of diseases in DO and MeSH. Then, the integrated disease glossary is used to annotate the metabolite-related diseases in HMDB. The associations between metabolites and diseases are established while DO serves as a unified representation of the disease in this study. Due to the expansion of the disease vocabulary, the naming characteristics of various diseases can be recognized in the process of disease name matching. The naming characteristics can be roughly divided into the following 5 cases.

- Singular, plural and possessive nouns in disease terms. The disease names may be singular or plural, but they all refer to the same disease term. For example, “Leukemia, Myelocytic” and “Leukemias, Myelocytic” represent the same disease term (DOID:8692); “Disease, Hodgkin’s” and “Disease, Hodgkin” represent the disease term (DOID:8692).

- Special symbols in disease terms. There may be some semantic irrelevant cases such as "-" or blank space in the disease terms. For example, “chickenpox” (DOID:8659) in DO is named as “Chicken Pox” (MESH:D002644) in MEDIC.
- Abbreviations in disease terms. Some disease names are abbreviated in the disease vocabulary. For example, both “Anorexia Nervosas and “AN” represent the same disease term (DOID:8689)
- Order of words in disease terms. In some disease names, the word order is reversed. For example, “type 1 diabetes mellitus” and “Diabetes Mellitus, Type 1” represent the same disease term (DOID:9744); “Neoplasm, Orbital” represents the same disease term (DOID:4143) as “Orbital Neoplasm”.
- Synonyms in disease terms. Some disease terms have synonyms. For example, “malignant tumor of lingual tonsil” and “malignant neoplasm of lingual tonsil” have the same meaning (DOID:8649).

The disease terms can be annotated to the maximum with the integrated disease glossary. A total of 1,406 associations between diseases and metabolites are obtained by matching the disease terms, including 600 human metabolites and 248 diseases.

### Disease-metabolite association matrix construction

**Fig. 9:**
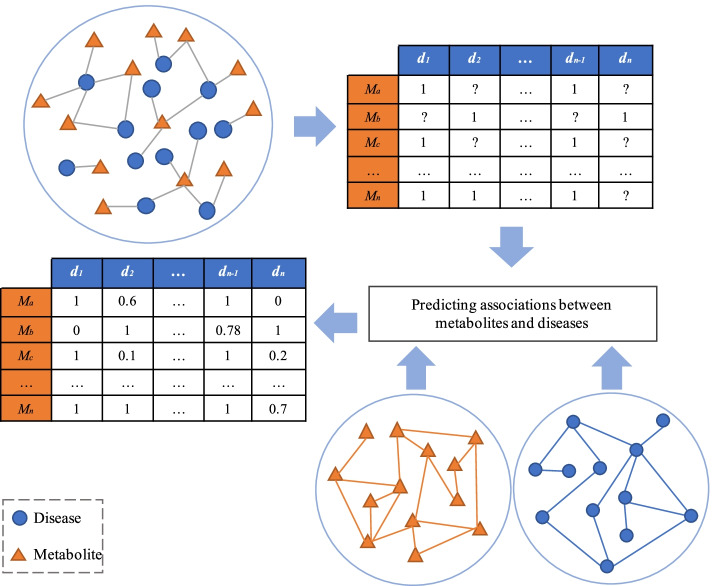
Schematic of the predictive associated matrix for diseases and metabolites

As one of the most successful technologies for recommender systems [39], collaborative filtering has been developed and improved over the past decade. In this study, we define associations between metabolites and diseases based on Collaborative Filtering and build the association matrix. The process of constructing the association matrix between diseases and metabolites is shown in Fig. 9. Firstly, the initial association matrix of diseases and metabolites is constructed based on the known associations between diseases and metabolites. Because it is a 0–1 matrix and its data is sparse, the unknown associations between metabolites and diseases can be calculated with disease similarities and metabolite similarities. As a result, we can get an association matrix of diseases and metabolites.

We firstly define the association matrix of diseases and metabolites as follows:

*Definition 1 Matrix MDR* = *[mdr(m,d)]*_*|M||D|*_* is the association matrix of diseases and metabolites, where |M| represents the number of disease-related metabolites and |D| is the number of metabolite-related diseases; mdr(m, d) is the association degree of metabolite m and disease d.*

Based on the known mapping between diseases and metabolites, we can get the initial association matrix *MDR*_*init*_, the initial association degree *mdr*_*init*_(*m*, *d*) of metabolite *m* and disease *d* can be defined as follows:

1 $$md{r}_{init}(m,d)=\left\{\begin{array}{ccc}\begin{array}{c}1\\ 0\end{array}& & \begin{array}{c}d\in {D}_{m}\\ otherwise\end{array}\end{array}\right.$$

where *D*_*m*_ represents the set of diseases related to metabolite *m*. If there exists any association between metabolite *m* and disease *d*, the association degree is 1; otherwise, it is 0.

It can be seen from Definition 1 that the number of diseases |*D*| is 248, the number of metabolites |*M*| is 600. But |*M*| ×|*D*| is much larger than the number of associations between diseases and metabolites. In other words, the initial association matrix is very sparse.

In order to solve the problem of data sparsity in the matrix, the disease similarities and metabolite similarities are used to complete the unknown associations in the matrix. In this paper, FNSemSim [40], which we previously developed, is used to calculate disease similarities. This method calculates disease similarities by a fused gene functional network composed of HumanNet [41] and FunCoup [42]. The results show that FNSemSim has a good performance for calculating similarities between diseases. Now, given the disease *d*_1_ and disease *d*_2_, if *d*_1_ ∈ *D* and *d*_2_ ∈ *D*, where *D* is the set of disease terms related to metabolites, then the similarity between disease *d*_1_ and disease *d*_2_ can be calculated by FNSemSim, denoted as *FNSim*(*d*_1_*, d*_2_).

We extract the text mining scores from STITCH [43] as the similarities of metabolites in this study. In the compound network of STITCH, the text association between compounds is quantified as a statistical score through corpus collection, word segmentation, name recognition, entity relationship integration. Here we extract these text scores between compounds. Based on the mapping between compounds and metabolites from HMDB, the text score between metabolite *m*_1_ and metabolite *m*_2_ can be denoted as *ST*(*m*_1_, *m*_2_). So the literature similarity between metabolite *m*_1_ and metabolite *m*_2_ is defined as follows:

2 $$MSim({m}_{1},{m}_{2})=\frac{{ST}_{max}-ST({m}_{1},{m}_{2})}{{ST}_{max}-{ST}_{min}}$$

where *ST*_max_ and *ST*_min_ represent the maximum and minimum scores among all metabolites respectively. The standardized score is considered as the similarity between metabolites. After obtaining similarities between diseases and metabolites, we complete the unknown associations in the initial matrix *MDR*_*init*_.

There are some diseases not associated with metabolite *m* in the matrix, but some connections between them and diseases associated with metabolite *m* can be built based on the disease similarities. So for ∀*m* ∈ *M*, *d* ∈ *D*, the association between metabolite *m* and disease *d* based on disease similarity is defined by Formula (3):

3 $$D{F}_{d,m}=\left\{\begin{array}{ccc}\begin{array}{c}md{r}_{init}(m,d)\\ MAX(FNSim(d,{d}_{i}))\end{array}& & \begin{array}{c}d\in {D}_{m}\\ {d}_{i}\in {D}_{m},d\notin {D}_{m}\end{array}\end{array}\right.$$

where *D*_m_ is the set of diseases related to metabolite *m*, and *D*_m_ ⊆ *D*, 1 ≤ *i* ≤|*D*_m_|. Disease *d*_i_ represents any disease in *D*_m_.

In the same way, the metabolite similarities are used to build connections between disease *d* and those metabolites not related to disease *d* in the matrix. So for ∀*d* ∈ *D*, *m* ∈ *M*, the association between metabolite *m* and disease *d* based on metabolite similarity is defined by Formula (4):

4 $$M{F}_{d,m}=\left\{\begin{array}{ccc}\begin{array}{c}md{r}_{init}(m,d)\\ MAX(MSim(m,{m}_{j}))\end{array}& & \begin{array}{c}m\in {M}_{d}\\ {m}_{j}\in {M}_{d},m\notin {M}_{d}\end{array}\end{array}\right.$$

where *M*_d_ is the set of metabolites related to disease *d*, and *M*_d_ ⊆ M, 1 ≤ *j* ≤|*M*_d_|. Disease *m*_j_ represents any disease in *M*_d_, which satisfies *mdr*_*init*_(*m*_*j*_, *d*) = 1.

Because *DF* and *MF* are independent of each other, the associations between diseases and metabolites in the matrix *MDR* can be defined as follows:

5 $$mdr(m,d)=1-(1-D{F}_{d,m})(1-M{F}_{d,m})$$

where *DF*_*d*,*m*_ and *MF*_*d*,*m*_ can be taken as the probability that disease *d* and metabolite *m* are related, so *mdr*(*m*, *d*) is regarded as the probability that at least one of the associations calculated based on different similarities exists. Finally, we can obtain an association matrix *MDR* about diseases and metabolites.

### Disease-related metabolite prediction

According to Definition 1, the matrix MDR contains the associations between diseases and metabolites. Therefore, disease-related metabolites can be classified based on the Latent Factor Model (LFM), and the connections between diseases and metabolites can be built by latent features.

In the matrix composed of diseases and metabolites, metabolites can be labeled according to the associations between diseases and metabolites. The potential associations between diseases and metabolites are determined by these labels. Therefore, the task of predicting potential associations between diseases and metabolites is to find the matrixes composed of diseases, disease-related metabolites and latent factors, and then complete this matrix of diseases and metabolites by reducing dimensions. The matrixes composed of diseases, disease-related metabolites and latent factors are defined as follows:

*Definition 2 Given the set of latent factors F, DLF* = *[dlf(d, f)]*_*|D||F|*_* is the association matrix of diseases and latent factors, MLF* = *[mlf(m, f)]*_*|M||F|*_* is the association matrix of latent factors and metabolites, where D is the set of diseases and M is the set of disease-related metabolites, m ∈ M, d ∈ D, f ∈ F.*

Matrix *DLF* and *MLF* can be seen as the representations of diseases and metabolites in the space of latent factors with |*F*| dimensions, respectively. So the matrixes defined in Definition 2 can be used to approximate the association matrix between diseases and metabolites. The approximate representation is defined as follows:

6 $$MD{R}^{*}=DLF*ML{F}^{T}$$

The purpose of figuring out the matrix MDR^*^ is to use the representation of diseases and metabolites in the latent factor space to maximize the approximation to the original association matrix *MDR*. Thus, the associations between diseases and metabolites can be predicted. In Formula (6), the predicted values of associations between disease *d* and metabolite *m* can be calculated as follows:

7 $$MD{R}_{m,d}^{*}={\sum }_{f\in F}^{F}DL{F}_{d,f}ML{F}_{m,f}$$

where *F* is the set of latent factors. For disease *d* and metabolite *m*, in order to approximate the predicted value MDR^*^_m,d_ to the actual value *MDR*_m,d_, the cost function can be defined as follows:

8 $$L={\sum }_{m\in M,d\in D}(MD{R}_{m,d}-MD{R}_{m,d}^{*}{)}^{2}+\frac{\lambda }{2}({\Vert D{F}_{d}\Vert }^{2}+{\Vert M{F}_{m}\Vert }^{2})$$

where *DF*_d_ and *MF*_m_ are respectively the vectors of disease *d* and metabolite *m* in the association matrix *MF* and *DF* with latent factors as dimensions. If the predicted value is closer to the actual one, the cost function *L* will be smaller; otherwise, it will be larger. To prevent overfitting, L2 regularization is performed on the cost function *L*. λ is the regularization parameter, which is used to weigh the regularization effect.

Here the stochastic gradient descent method is used to optimize the cost function. After the cost function expanded, the direction of the fastest descent is determined by calculating the partial derivatives of *DLF*_d,f_ and *MLF*_m,f_. Their gradient formulas are expressed as follows:

9 $$\frac{\partial L}{\partial DL{F}_{d,f}}=-2(MD{R}_{m,d}-MD{R}_{m,d}^{*})ML{F}_{m,f}+\lambda DL{F}_{d,f}$$

10 $$\frac{\partial L}{\partial ML{F}_{m,f}}=-2(MD{R}_{m,d}-MD{R}_{m,d}^{*})DL{F}_{d,f}+\lambda ML{F}_{m,f}$$

Then, the values in the matrix *MF* and *DF* are trained based on the stochastic gradient descent method. The recursive formulas are defined as follows:

11 $$DL{F}_{d,f}=DL{F}_{d,f}+\alpha \frac{\partial L}{\partial DL{F}_{d,f}}$$

12 $$ML{F}_{m,f}=ML{F}_{m,f}+\alpha \frac{\partial L}{\partial ML{F}_{m,f}}$$

where α is the learning rate. The parameters are constantly optimized through iterative calculation until the approximate matrix converges. So for ∀*d* ∈ *D*, *m* ∈ *M*, the association degree between disease *d* and metabolite *m* is defined as follows:

13 $$MD{R}_{m,d}=\left\{\begin{array}{ccc}\begin{array}{c}1\\ MD{R}_{m,d}^{*}\end{array}& & \begin{array}{c}\text{if }md{r}_{init}(m,d)=1\\ otherwise\end{array}\end{array}\right.$$

where MDR^*^_m,d_ is the potential association between disease *d* and metabolite *m*.

## Data Availability

DLMPM is implemented using a combination of Java and scala, and it is freely available with all data sets by https://github.com/wyt-nwpu/DLMPM.

## Abbreviations

AUC: Area under the receiver operating characteristic curve
LOOCV: Leave One Out Cross Validation
SVD: Singular Value Decomposition
DO: Disease Ontology
HMDB: Human Metabolome Database
STITCH: Search tool for interactions of chemicals
MeSH: Medical Subject Headings
MEDIC: Merged Disease Vocabulary
CTD: Comparative Toxicogenomics Database
